# Inappropriate Polypharmacy and the Need for Comprehensive Medication Management Service

**DOI:** 10.18295/squmj.9.2023.055

**Published:** 2024-05-27

**Authors:** Juhaina S. Al-Maqbali, Ibrahim Al-Zakwani

**Affiliations:** Department of Pharmacology & Clinical Pharmacy, College of Medicine and Health Science, Sultan Qaboos University, Muscat, Oman; Department of Pharmacy, Sultan Qaboos University Hospital, University Medical City, Muscat, Oman

Polypharmacy is commonly defined as taking 5 or more medications at once by a patient.^1^^,^^2^ Even though polypharmacy is observed in the majority of age groups, at a prevalence rate of around 37%, it is, however, more prevalent (54%) in the elderly (>65 years). Polypharmacy is linked to negative outcomes including increasing morbidities and mortality, especially in the elderly, and at Sultan Qaboos University Hospital (SQUH), Muscat, Oman, it was reported to be an independent risk factor for delirium development during hospitalisation.^3^^,^^4^ Polypharmacy is also known to be a major risk factor for adverse drug reactions (ADRs), drug-drug interactions, and hospital re-admissions.

A report of polypharmacy interventions has been published that is associated with improvements in patients’ health outcomes including quality of life, disease control as well as a reduction in hospital costs.^5^ This includes the availability of clinical pharmacy service, where clinical pharmacists in multidisciplinary care teams of various models, play a fundamental role in enhancing patient health outcomes and reducing the economic burden.^6^^,^^7^ At SQUH, clinical pharmacy services have been provided to most but not all specialties since the inception of the hospital and the pharmaceutical interventions are intended to care for patients from admission until discharge.^6^–^9^ The pharmaceutical interventions were able to prevent a wide range of medication errors and inappropriate polypharmacy which led to a reported annual cost avoidance of approximately US$440,000.^8^ Deleting or omitting a medication from a patient list, especially if it's contributing to inappropriate polypharmacy is considered a fundamental category of pharmaceutical interventions provided by the clinical pharmacists at SQUH and its highly acknowledged by the healthcare providers.^9^ Furthermore, the clinical pharmacy service provided at SQUH was further developed to an emerging concept called ‘bundle care service’ that provides a whole bundle of care to the patients on admission, which includes medication history/reconciliation on admission, pharmaceutical interventions, discharge medication review and counselling during the hospital stay or upon discharge to ensure a proper transition of care.^7^^,^^10^ It has been reported that the full bundle of care is prioritised to patients with more than one comorbidity or to those on polypharmacy and to not all admitted patients, mainly because of the insufficient number of clinical pharmacists available at SQUH.

The need for a sustained pharmacy practice model that contributes to the institutional and governmental strategic plans for reducing healthcare costs and improving clinical outcomes is frequently recommended, which ensures an early follow-up review post-hospitalisation.^11^ An example of this is the new concept of comprehensive medication management (CMM) service that guarantees each patient’s medications are reviewed to determine their appropriateness, effectiveness and safety given their complex comorbidities and other prescribed medications. The medications should be taken by the patient as intended during a continuous follow-up plan that starts at the primary care or post-hospitalisation.^12^ Unlike other pharmaceutical models, CMM service is delivered in the form of a continuous patient-centred model with a holistic approach, by clinical pharmacists working with the patient, physicians and other members of the healthcare team.^13^ This leads in return to continuous prevention of inappropriate polypharmacy and other pharmaceutical care issues. However, the insufficient number of clinical pharmacists available at SQUH is still the main limitation for the optimal implementation of those services. The decision of implementing these new services at SQUH should consequently be guided by the return on investment and cost-benefit analyses. This concept was first recommended by the American College of Clinical Pharmacy and its clinical and economic impact on primary care was widely and positively supported by evidence-based literature.^14^

At the start of CMM service, the triple aim was broadly agreed upon to optimise the healthcare system performance, which enhances patient experience, improves population health and reduces costs. In the past decade, a growing body of evidence has documented the benefits of moving CMM service from double and triple aim to quadruple aim, including patient satisfaction and impact on provider (e.g. pharmacists and physicians) work-life balance together with the earlier mentioned benefits of healthcare utilisation and clinical outcomes, through demonstrating documentation processes and monitoring surveys.^15^ This movement from triple aim to quadruple aim was affected by physicians and other members of the healthcare workforce reporting a widespread burnout and dissatisfaction, which in turn may have impacted health outcomes and increased costs. CMM service is widely provided in primary care and assisted in shaping the CMM service for other types of care, depending on organisational needs, availability of resources and differences in pharmacy practice models. CMM was later improved to handle the secondary and tertiary care level.^16^ In addition, measurable criteria to identify patients and areas for practicing CMM services have been developed to help prioritise patients who would benefit most from clinical pharmacist interventions.^17^ These include: classifying the patient’ medication list into complex, risky or costly medication as well as innovations to establish physician-pharmacist collaborative relationships to aid pharmacists practicing CMM using various strategies that are mainly around the concept of physicians accepting pharmacist interventions.^18^ Although recent studies published on clinical pharmacy services at SQUH have proven that staff shortage in this service can lead to less than optimum expectations, they have also documented a high acceptance rate among other healthcare providers about their interventions that lead to better health outcomes.^7^–^9^ This might be an opportunity for SQUH to evaluate the implementation of CMM services by prioritising healthcare conditions that are likely to be associated with inappropriate polypharmacy. These could include chronic diseases such as heart failure, diabetes mellitus and hypertension. Additionally, other categories of patients could be added to the referral service. This might prove to be an efficient and cost-effective service at SQUH.
